# *TSP-1*-1223 A/G Polymorphism as a Potential Predictor of the Recurrence Risk of Bladder Cancer in a Chinese Population

**DOI:** 10.1155/2013/473242

**Published:** 2013-12-03

**Authors:** Xiao Yang, Pengchao Li, Xuejian Yang, Chao Qin, Qiang Cao, Zhengdong Zhang, Meilin Wang, Hongzhou Cai, Jinbao Gu, Jun Tao, Min Gu, Qiang Lu, Changjun Yin

**Affiliations:** ^1^Department of Urology, The First Affiliated Hospital of Nanjing Medical University, Nanjing 210029, China; ^2^Cancer Center of Nanjing Medical University, Department of Molecular & Genetic Toxicology, Nanjing Medical University, 140 Hanzhong Road, Nanjing 210029, China

## Abstract

*Backgrounds*. TSP-1 is a glycoprotein that functions in the biology of bladder cancer. We investigated the relationship between the distribution of *TSP-1*-1223 A/G polymorphism (rs2169830) and the clinical characteristics of bladder cancer. *Materials and Methods*. TaqMan assay was performed to determine the genotype of 609 cases and 670 control subjects in a Chinese population. Logistic regression was used to assess the association between the polymorphism and the risk of bladder cancer. Quantitative real-time polymerase chain reaction was performed to determine *TSP-1* mRNA expression. Survival curves were generated using the Kaplan-Meier method. *Results*. No significant differences were detected in the genotype frequencies of healthy control subjects and patients with bladder cancer. By contrast, the time until the first recurrence differed significantly between genotypes (*P* = 0.017). The expression of *TSP-1* mRNA in bladder cancer tissues was lower in patients with an AG genotype than in those with an AA genotype. The lowest expression was observed in patients with a GG genotype. *Conclusions*. In conclusion, *TSP-1*-1223 A/G polymorphism may contribute to the recurrence of bladder cancer in Chinese population.

## 1. Introduction

Bladder cancer is the second most common genitourinary malignancy and the sixth most common cancer in the world [1]. In China, the incidence of bladder cancer continues to increase [2]. The incidence of bladder cancer also increases with age, and the peak is reached approximately at the age of 60 years; bladder cancer is three times more common in men than in women [3]. Among the newly diagnosed cases of transitional cell carcinomas, approximately 75% to 80% of these cases present superficial tumours; 50% to 70% of these superficial tumours relapse within five years; and roughly 10% to 20% progress to a more aggressive disease [4]. Bladder cancer is a multifactorial disease mediated by genetic abnormalities, environmental factors, and chronic irritation [5]. Although many individuals are exposed to these risk factors, only a fraction of exposed individuals develop bladder cancer in their lifetime, suggesting that genetic variations may participate in bladder carcinogenesis.

Thrombospondin-1 (TSP-1) is an adhesive glycoprotein with a size of 450 kD initially discovered in platelets, where TSP-1 is sequestered in a platelet *α*-granule [6]. TSP-1 has been implicated in regulating numerous biological activities, including cell adhesion, cell migration, proliferation, angiogenesis, inflammation, and wound healing [7–12]. TSP-1 also elicits different effects on tumour growth and progression depending on the tumour type, leading to cancer progression and inhibition in different instances [11–18]. In bladder cancer, low TSP-1 expression is significantly associated with an increased risk of disease recurrence and decreased overall survival [17, 18].

Genetic factors have a critical function in initiating cancer. Studies have been conducted to assess the association between polymorphisms in candidate genes and bladder cancer risk [19]. To date, genetic polymorphisms in *TSP-1* gene are possibly associated with coronary artery disease and myocardial infarction [20, 21]. The transcriptional initiation site of the *TSP-1* gene was identified by Laherty et al. [22]. *TSP-1*-1223 A/G polymorphism is located in the 5′ near-gene region of the *TSP-1* gene, which may influence the transcriptional activity of *TSP-1*. Furthermore, *TSP-1*-1223 A/G polymorphism may participate in the aetiology and development of bladder cancer.

In the present study, *TSP-1*-1223 A/G polymorphism (rs2169830) may be involved in the predisposition to develop bladder cancer. To validate this hypothesis, we genotyped the polymorphism and determined its association with the risk of bladder cancer in our ongoing, hospital-based, case-control study in a Chinese population.

## 2. Materials and Methods

### 2.1. Ethics Statement

The study was approved by the Institutional Review Board of the First Affiliated Hospital of Nanjing Medical University, Nanjing, China. During recruitment, written informed consent was obtained from all of the participants involved in this study.

### 2.2. Patients and Controls

The present study included 609 patients with bladder cancer and 670 age-matched control subjects from Han population living in Jiangsu and Anhui provinces in Eastern China. The patients with bladder cancer were recruited from July 2006 to July 2012 in the Department of Urology, the First Affiliated Hospital of Nanjing Medical University. The patients were excluded in this study according to the following: previous cancer, metastasised cancer from other or unknown origins, and previously subjected to radiotherapy or chemotherapy. Diagnosis of bladder cancer was confirmed by histopathological analysis. Cancer-free control individuals were frequency matched with the cancer patients in terms of age (±5 years) and gender. We also ensured that these control subjects were genetically unrelated to the cases. All of the control subjects were recruited from healthy individuals who were scheduled for a physical examination in the outpatient department at the same hospital. The control subjects were excluded if they manifested symptoms of bladder cancer, such as haematuria. Prior to recruitment, all of the subjects were personally interviewed to collect demographic data and clinical characteristics, including age, gender, race, tobacco use, alcohol use, and self-reported family history of cancer. According to the tumour, node, and metastasis classification of cancer stages (2002 International Union Against Cancer), the clinical stage at the time of diagnosis was classified into two subgroups: nonmuscle invasive group (pTa-pT1) and muscle invasive group (pT2-pT4). According to histopathological grade (WHO 1973, grading of urothelial papilloma), the patients were classified into three subgroups: grades 1, 2, and 3. Individuals who smoked daily for >1 year were defined as smokers and the rest were considered as nonsmokers. Individuals who drank alcohol at least three times per week for more than 6 months were defined as drinkers and the rest were considered as nondrinkers.

For survival analysis, 260 patients were followed up. Among these follow-up cases, 24 (24/260, 9.2%) were excluded because of incomplete follow-up data. Survival time was calculated from the date of confirmed diagnosis until the date of the last follow-up or recurrence. The date of recurrence was obtained from inpatient and outpatient records or from patients' families via follow-up telephone calls. The patients who did not suffer from recurrence on the last follow-up date were considered as non-recurrent.

### 2.3. DNA Extraction and Polymorphism Genotyping

Genomic DNA of each individual was extracted from 150 *μ*L of EDTA-anticoagulated peripheral blood samples by using a DNA extraction kit (Tiangen Biotech, Beijing, China) according to the manufacturer's instructions. The *TSP-1*-1223 A/G polymorphism was genotyped using TaqMan single nucleotide polymorphism (SNP) genotyping assay (Applied Biosystems, Foster City, CA, USA). SDS 2.4 software was used for allelic discrimination. The primers, probes, and reaction conditions of each SNP are available upon request. For quality control, four negative controls were included in each plate, and 5% of the samples were randomly selected for repeated genotyping to verify the results; all of the results were 100% consistent.

### 2.4. Quantitative Real-Time PCR

Total RNA from 66 bladder cancer tissues was extracted using Trizol reagent (Invitrogen, Carlsbad, CA) according to the manufacturer's protocol. Total RNA (1 *μ*g) was used for cDNA synthesis with oligo-dT primers (Invitrogen, Karlsruhe, Germany) and superscript II reverse transcriptase (Takara Bio, Shiga, Japan). PCR was performed using PCR Master (Roche, Mannheim, Germany) with the following primers: for *TSP-1* mRNA, 5′-ACTGTCCATTCCATTACAACCCAGC-3′ (forward) and 5′-TGTCACACTGATCTCCAACCCCATCCA-3′ (reverse); and for **β*-actin*, 5′-ACTGGAACGGTGAAGGTGAC-3′ (forward) and 5′-AGAGAAGTGGGGTGGCTTTT-3′ (reverse). Fold changes were normalised based on **β*-actin* expression, and each assay was conducted in a 384-well ABI 7900HT real-time PCR system (Applied Biosystems, Foster City, CA, USA). This procedure was performed in triplicate.

### 2.5. Statistics Analysis

The frequency distributions of the selected demographic variables as well as each allele and genotype of the *TSP-1*-1223 A/G polymorphism between the cases and the control subjects were evaluated using *χ* ^2^-type distribution. Hardy-Weinberg equilibrium (HWE) was determined using a goodness-of-fit *χ* ^2^ test. Unconditional univariate and multivariate logistic regression analyses were conducted to calculate the crude and adjusted odds ratios (ORs) and 95% confidence intervals (CIs) to determine the risk of bladder cancer. Interaction was investigated using a multiplicative interaction term included in the multivariate model. To investigate potential interactions between the polymorphism and tobacco smoking, we assessed a multiplicative gene-environment interaction by logistic regression analysis, including the main effect variables and their product terms. Hazard ratios (HRs) and 95% CIs of the HRs were derived from univariate and multivariate Cox proportional hazard models. Survival curves were generated using the Kaplan-Meier method and compared using the log-rank-Mantel-Cox test. All of the analyses were carried out using SPSS 13.0. A two-sided *P* value <0.05 represented a statistically significant result.

## 3. Results

### 3.1. Characteristics of the Study Population

The frequency distributions of the selected characteristics of the cases and the control subjects are shown in Table 1. The cases and the control subjects were adequately matched in terms of age and gender (*P* = 0.486 for age and *P* = 0.091 for gender). A higher number of smokers were found among the cases than the control subjects (47.9% versus 36.4%; *P* < 0.001). No significant difference in drinking status was found between the cases and the control subjects (*P* = 0.196). In addition, the frequency of the first-degree relatives with cancer was higher in the cases than in the control subjects (27.8% versus 7.2%; *P* < 0.001). These variables were further adjusted in the multivariate logistic regression analysis to assess the main effect of the *TSP-1*-1223 A/G polymorphism on bladder cancer risk. The clinicopathological characteristics of 609 cases with bladder cancer are listed in Table 1.

### 3.2. Association between *TSP-1*-1223 A/G Polymorphism and Risk of Bladder Cancer

The genotype and allele frequency distributions of the *TSP-1*-1223 A/G polymorphism among the cases and the control subjects as well as their associations with the risk of bladder cancer are presented in Table 2. The genotype frequencies in the control subjects are consistent with those of HWE (*P* = 0.851). The frequencies of AA, AG, and GG genotypes were 47.1%, 40.2%, and 12.6% among the cases and 43.9%, 44.5%, and 11.6% among the control subjects, respectively (*P* = 0.307). No correlation was observed between *TSP-1*-1223 A/G polymorphism and the risk of bladder cancer. We further assessed the effect of *TSP-1*-1223 A/G polymorphism on the risk of bladder cancer risk stratified by age, gender, smoking status, drinking status, self-reported family history of cancer, and tumour grade and tumour stage (Tables 3, 4, and 5). Logistic regression analysis also showed no association between *TSP-1*-1223 A/G polymorphism and bladder cancer risk.

### 3.3. Interaction between *TSP-1*-1223 A/G Polymorphism and Tobacco Smoking

A significantly increased risk was observed in smokers with AA/AG genotypes compared with nonsmokers exhibiting AA/AG genotypes (*P* = 0.001; ORs = 1.58; 95% CI = 1.20 to 2.10; Table 6). We then evaluated whether or not an interaction between smoking status and *TSP-1*-1223 A/G polymorphism status occurs. We did not observe a multiplicative interaction effect between polymorphism and smoking status (*P* = 0.823; Table 6).

### 3.4. Effects of *TSP-1*-1223 A/G Polymorphism and Bladder Cancer Recurrence

We further investigated whether or not *TSP-1*-1223 A/G polymorphism is associated with the recurrence of bladder cancer. The demographic and clinical characteristics at primary diagnosis and the genotype distribution in patients with bladder cancer are shown in Table 7. The median follow-up duration for the 236 patients was 19 months (range, 1 month to 68 months). The mean time-to-recurrence was 20.8 months (95% CI = 17.2 to 24.3 months). Kaplan-Meier curves showed statistical difference between *TSP-1*-1223 A/G polymorphism and time-to-recurrence in bladder cancer (AA 20.5 months, 95% CI = 14.7 to 26.3; AG 22.2 months, 95% CI 15.6 to 28.7; GG 18.8 months, 95% CI 12.7–25.0; log-rank test, *P* = 0.017, Figure 2). Compared with AA + AG genotypes, the GG genotype exhibited a significant recurrence risk of bladder cancer (HR = 2.07, 95% CI = 1.23 to 3.49, *P* = 0.006), and the recurrence risk was more prominent among patients with the GG genotype (HR = 2.63, 95% CI = 1.43 to 4.83, *P* = 0.002). The cases exhibiting AG + GG genotypes were also associated with an increased recurrence risk compared with the AA genotype (HR = 1.95, 95% CI = 1.20 to 3.19, *P* = 0.007; Table 8).

### 3.5. Association between *TSP-1*-1223 A/G Polymorphism and *TSP-1* mRNA Expression

**Figure 1:**
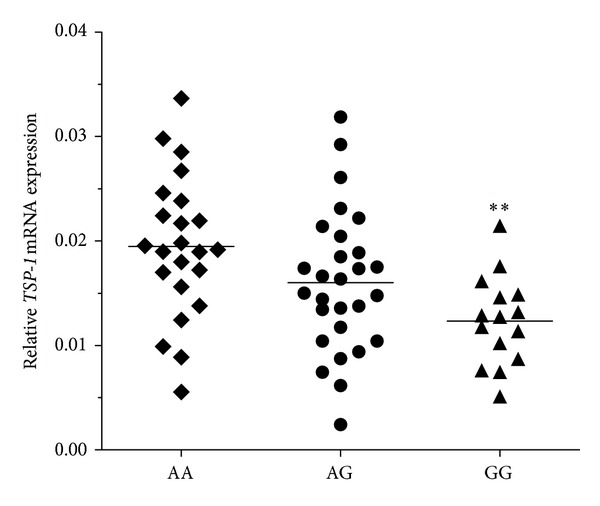
Analysis of *TSP-1* mRNA expression levels in three groups of bladder cancer tissue samples with mean values (horizontal lines, mean values). **P < 0.01 relative to AA genotype.

**Figure 2:**
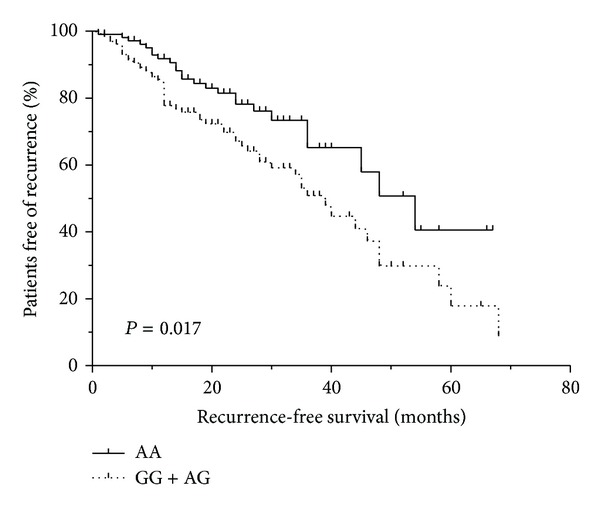
Kaplan-Meier survival curves for recurrence among bladder cancer cases based on *TSP-1*-1223 A/G polymorphism genotypes. *P* values were calculated by log-rank tests.

To evaluate the association between *TSP-1*-1223 A/G polymorphism and recurrence of bladder cancer, we examined whether or not the *TSP-1*-1223 A/G polymorphism was associated with an altered *TSP-1* mRNA expression. No significant difference was observed in the relative *TSP-1* mRNA expression level between patients with AA (*n* = 23) and AG (*n* = 28) genotypes (*P* = 0.103). The patients with the GG genotype exhibited lower *TSP-1* mRNA expression levels than those with AA genotype (*n* = 15; *P* = 0.002), although a significant overlap among the three groups was observed (Figure 1).

## 4. Discussion

In the present study, the association between -1223 A/G polymorphism in *TSP-1* gene (rs2169830) and risk of bladder cancer was investigated. The results do not indicate that the genotypes of the *TSP-1*-1223 A/G polymorphism are associated with an increased bladder cancer incidence. However, the time-to-recurrence was significantly shorter in G allele carriers than that in individuals with a homozygous AA genotype. Patients with the AG/GG genotypes have 1.95 times greater risk of the recurrence of bladder cancer than those with the AA genotype (*P* = 0.007). Furthermore, individuals with the GG genotype exhibited a higher risk of recurrence than those with the AA genotype (OR = 2.63, *P* = 0.002). To the best of our knowledge, this study is the first to investigate the function of the *TSP-1*-1223 A/G polymorphism in the aetiology of bladder cancer.

TSP-1 has been implicated in tumour growth and progression by regulating cell adhesion [23], motility [24], proliferation [25], and angiogenesis [26]. As a multifunctional protein involved in tumour growth regulation, TSP-1 elicits an inhibitory effect on the growth of various malignancies such as melanoma [27], prostate cancer [28], cutaneous squamous cell carcinoma [29], and glioblastoma [30]. Strong evidence has also suggested that TSP-1 may stimulate lung cancer [31] and breast cancer [32].

A previous study identified that TSP-1, secreted by normal urothelium cells, is responsible for antiangiogenic activity [33]. Angiogenesis is associated with an increased recurrence risk of bladder cancer [34]. The downregulation of TSP-1 in bladder cancer is considered as a primary factor contributing to specific changes, for instance, from an antiangiogenic to an angiogenic phenotype during cancer development and recurrence [17, 18, 33].

Most of the *TSP-1* SNPs have only been observed in cardiac disease [20, 21]; no studies have been shown to provide a relationship between *TSP-1* gene polymorphism and cancer. In our study, the GG/AG genotypes of the *TSP-1*-1223 A/G polymorphism in bladder cancer were associated with a shorter time-to-recurrence than the AA genotype. Individuals carrying the lower production genotype of *TSP-1*-1223 A/G polymorphism possibly possess an enhanced ability to promote the recurrence of bladder cancer.

Using the program TFSEARCH, we found that the G allele of *TSP-1*-1223 A/G polymorphism was located in the consensus DNA sequence TGTGGT. The DNA sequence was a potential transcription regulation region, which can be recognised by AML1-a [35, 36]. However, any transcriptional factor was not bound to the sequence TGTGAT containing the A allele of the *TSP-1*-1223 A/G polymorphism. Furthermore, AML1-a participates as a transcription inhibitor by suppressing the transcriptional activation of AML1-b [37]. Thus, we cautiously speculated that AML1-a binds to the consensus sequence (TGTGGT) of the G allele in the *TSP-1*-1223 A/G polymorphism, thereby suppressing the transcription of *TSP-1*.

A previous study revealed that TSP-1 can suppress VEGF mobilisation from the extracellular matrix by inhibiting the activity of MMP-9 [38]. TSP-1 can also inhibit the activity of VEGF by interacting directly with VEGF [39]. The possible mechanism between angiogenesis and bladder cancer recurrence has been related to an increased VEGF expression level, which serves as a major factor in angiogenesis [40]. The increased expression level of VEGF was implicated in the pathogenesis of bladder cancer recurrence by promoting the growth and implantation of a bladder cancer cell via angiogenesis [41]. Conversely, decreased TSP-1 may be associated with an increased risk of bladder cancer recurrence via the inhibitory effect of TSP-1 on VEGF-mediated tumour angiogenesis.

In the current study, the expression level of *TSP-1* mRNA in bladder cancer samples was lower in patients with heterozygous G allele than those with homozygous A allele. The expression level of *TSP-1* mRNA was the lowest in individuals with homozygous G allele. As such, the shorter time-to-recurrence in patients with bladder cancer exhibiting a G allele may be attributed to a decreased *TSP-1* expression, which has been associated with an increased risk of disease recurrence and a decreased overall survival by promoting tumour neovascularization [17, 18, 34]. Our results partly supported our hypothesis; however, further functional experiments should be conducted to validate the specific mechanism.

Several potential limitations are observed in the present study. First, our sample size was relatively small, which may limit the statistical power of our study, particularly for gene-environment interaction analyses. Second, our study was a hospital-based case-control study; hence, we cannot rule out the possibility of selection bias for subjects who may have been associated with a particular genotype.

In conclusion, our study shows that G allele in *TSP-1*-1223 A/G polymorphism may modulate the risk of recurrence in bladder cancer. A lack of association between *TSP-1*-1223 A/G polymorphism and risk of bladder cancer was observed in our population. Nevertheless, a possible function of this polymorphism in other cancers or in other bladder cancer populations should be considered. Hence, additional studies should be conducted to confirm the specific function of *TSP-1*-1223 A/G polymorphism in bladder cancer development.

## Figures and Tables

**Table 1:** Frequency distribution of selected variables of the bladder cancer cases and controls.

Variables	Cases (*n* = 609)		Controls (*n* = 670)		*P* ^a^
	*n*	%	*n*	%	
Age (year)					
<65	289	47.5	331	49.4	0.486
≥65	320	52.5	339	50.6	
Sex					
Male	484	79.5	506	75.5	0.091
Female	125	20.5	164	24.5	
Smoking status					
Never	317	52.1	426	63.6	<0.001
Ever	292	47.9	244	36.4	
Drinking status					
No	418	68.6	482	71.9	0.196
Yes	191	31.4	188	28.1	
Family history of cancer					
No	440	72.2	622	92.8	<0.001
Yes	169	27.8	48	7.2	
Tumor stage					
Nonmuscle invasive	466	76.5			
Muscle invasive	143	23.5			
Tumor grade					
Grade 1	298	48.9			
Grade 2	194	31.9			
Grade 3	117	19.2			

^a^Student's *t*-test for age distribution between the cases and controls; two-sided *χ* ^2^ test for other selected variables between the cases and controls.

**Table 2:** Genotype and allele frequencies of the *TSP-1*-1223 A/G polymorphism among the bladder cancer cases and controls.

Genotypes	Cases (*n* = 609)		Controls (*n* = 670)		*P* ^a^	Crude OR (95% CI)	*P* ^b^	Adjusted OR (95% CI)^b^
	*n*	%	*n*	%				
AA	287	47.1	294	43.9		1.00 (reference)		1.00 (reference)
AG	245	40.2	298	44.5	0.151	0.84 (0.66–1.07)	0.083	0.80 (0.62–1.03)
GG	77	12.6	78	11.6	0.951	1.01 (0.70–1.46)	0.603	1.11 (0.75–1.63)

AA	287	47.1	294	43.9		1.00 (reference)		1.00 (reference)
AG + GG	322	52.9	376	56.1	0.244	0.88 (0.70–1.10)	0.191	0.85 (0.67–1.08)

AG + AA	532	87.4	592	86.6		1.00 (reference)		1.00 (reference)
GG	77	12.6	78	13.4	0.583	1.10 (0.77–1.56)	0.330	1.20 (0.83–1.72)

A allele	819	67.2	886	66.1				
G allele	399	32.8	454	33.9	0.548			

CI: confidence interval; OR: odds ratio.

^a^Two-sided *χ* ^2^ test for either genotype distributions or allele frequencies between the cases and controls.

^b^Adjusted for age, gender, smoking, drinking status, and family history of cancer in logistic regression model.

**Table 3:** Stratification analyses between *TSP-1*-1223 A/G polymorphism and risk of bladder cancer.

Variables		Genotypes (cases/controls)							
	Cases/controls	AA + AG		GG		*P* ^a^	Crude OR (95% CI)	*P* ^b^	Adjusted OR (95% CI)^b^
		*n*	%	*n*	%				
*Total *									
Age (year)									
<65	289/331	243/289	84.1/87.3	46/42	15.9/12.7	0.251	1.30 (0.81–2.10)	0.128	1.38 (0.86–2.25)
≥65	320/339	289/303	90.3/89.4	31/36	9.7/10.6	0.692	0.90 (0.53–1.55)	0.719	0.94 (0.55–1.61)
Sex									
Male	484/506	423/457	87.4/90.3	61/49	12.6/9.7	0.144	1.34 (0.89–2.05)	0.127	1.39 (0.91–2.13)
Female	125/164	109/135	87.2/82.3	16/29	12.8/17.7	0.257	0.68 (0.33–1.38)	0.495	0.78 (0.38–1.60)
Smoking status									
Never	317/426	278/377	87.7/88.5	39/49	12.3/11.5	0.738	1.08 (0.67–1.73)	0.381	1.25 (0.76–2.05)
Ever	292/244	254/215	86.6/88.1	38/29	13.4/11.9	0.694	1.11 (0.64–1.93)	0.747	1.10 (0.63–1.91)
Drinking status									
No	418/482	367/423	87.8/87.8	51/59	12.2/12.2	0.986	1.00 (0.65–1.52)	0.644	1.11 (0.71–1.73)
Yes	191/188	165/169	86.4/89.9	26/19	13.6/10.1	0.291	1.40 (0.71–2.79)	0.396	1.34 (0.68–2.64)
Family history of cancer									
No	440/622	385/547	87.5/87.9	55/75	12.5/12.1	0.829	1.04 (0.70–1.53)	0.558	1.12 (0.76–1.65)
Yes	169/48	147/45	87.0/93.7	22/3	13.0/6.3	0.195	2.24 (0.63–12.2)	0.284	2.02 (0.56–7.26)

CI: confidence interval; OR: odds ratio.

^a^Two-sided *χ* ^2^ test for either genotype distributions or allele frequencies between the cases and controls.

^b^Adjusted for age, gender, smoking, drinking status, and family history of cancer in logistic regression model.

**Table 4:** The associations between *TSP-1*-1223 A/G polymorphism and the development of bladder cancer.

Variables	Genotypes				*P* ^a^	Crude OR (95% CI)	*P* ^b^	Adjusted OR (95% CI)^b^
	AA + AG		GG					
	*n*	%	*n*	%				
Controls (*n* = 670)	592	88.4	78	11.6		1.00 (reference)		1.00 (reference)
Cases (*n* = 609)								
Tumor stage								
Nonmuscle invasive	407	87.3	59	12.7	0.604	1.10 (0.75–1.60)	0.341	1.21 (0.82–1.79)
Muscle invasive	125	87.4	18	12.6	0.750	1.09 (0.59–1.92)	0.488	1.23 (0.68–2.22)
Tumor grade								
Grade 1	264	88.6	34	11.4	0.917	0.98 (0.62–1.52)	0.842	1.05 (0.67–1.65)
Grade 2	168	86.6	26	13.4	0.507	1.17 (0.70–1.92)	0.271	1.35 (0.79–2.30)
Grade 3	100	85.5	17	14.5	0.376	1.29 (0.69–2.31)	0.158	1.58 (0.84–2.97)
Number								
Single	384	88.1	52	11.9	0.886	1.03 (0.69–1.52)	0.550	1.13 (0.76–1.69)
Multiple	148	85.5	25	14.5	0.315	1.28 (0.75–2.12)	0.160	1.47 (0.86–2.50)
Size								
<3 cm	345	88.2	46	11.8	0.952	1.01 (0.67–1.51)	0.800	1.06 (0.70–1.60)
≥3 cm	187	85.8	31	14.2	0.314	1.26 (0.78–2.00)	0.074	1.56 (0.96–2.55)

CI: confidence interval; OR: odds ratio.

^a^Two-sided *χ* ^2^ test for either genotype distributions or allele frequencies between the cases and controls.

^b^Adjusted for age, gender, smoking, drinking status, and family history of cancer in logistic regression model.

**Table 5:** *TSP-1*-1223 A/G polymorphism and clinicopathological characteristics in patients with bladder cancer.

Clinicopathological characteristics	Genotypes *n* (%)		*P* ^a^	Adjusted OR (95% CI)^a^
	AA + AG	GG		
Tumor stage				
Nonmuscle invasive	407 (87.3)	59 (12.7)		1.00 (reference)
Muscle invasive	125 (87.4)	18 (12.6)	0.979	1.01 (0.57–1.78)
Tumor grade				
Grade 1	264 (88.6)	34 (11.4)		1.00 (reference)
Grade 2	168 (86.6)	26 (13.4)	0.440	1.26 (0.71–2.23)
Grade 3	100 (85.5)	17 (14.5)	0.294	1.43 (0.73–2.79)
Number				
Single	384 (88.1)	52 (11.9)		1.00 (reference)
Multiple	148 (85.5)	25 (14.5)	0.433	1.23 (0.73–2.07)
Size				
<3 cm	345 (88.2)	46 (11.8)		1.00 (reference)
≥3 cm	187 (85.8)	31 (14.2)	0.367	1.24 (0.74–2.08)

CI: confidence interval; OR: odds ratio.

^a^Adjusted for age, gender, smoking, drinking status, and family history of cancer in logistic regression model.

**Table 6:** Interaction analyses of the *TSP-1*-1223 A/G polymorphism and tobacco smoking.

Smoking status	Genotypes	Cases		Controls		*P* ^a^	Crude OR (95% CI)^a^	*P* ^b^	Adjusted OR (95% CI)^b^
		*n*	%	*n*	%				
Nonsmokers	AA + AG	278	45.7	377	56.3		1.00 (reference)		1.00 (reference)
Nonsmokers	GG	39	6.4	49	7.3	0.738	1.08 (0.67–1.73)	0.381	1.25 (0.76–2.05)
Smokers	AA + AG	254	41.7	215	32.1	<0.001	1.60 (1.25–2.05)	0.001	1.58 (1.20–2.10)
Smokers	GG	38	6.2	29	4.3	0.025	1.78 (1.04–3.06)	0.158	1.52 (0.85–2.73)
*P*_Interaction_ (multiplicative)									0.823

CI: confidence interval; OR: odds ratio.

^a^Two-sided *χ* ^2^ test for either genotype distributions or allele frequencies between the cases and controls.

^b^Adjusted for age, gender, smoking, drinking status, and family history of cancer in logistic regression model.

**Table 7:** Demographic and clinical characteristics at primary diagnosis and genotype distribution in patients with bladder cancer.

	All	Genotypes			*P*
		AA	AG	GG	
*N* (%)	236	104 (44.1)	91 (38.6)	41 (17.4)	
Sex (M/F)	190/46	87/17	73/18	30/11	0.356
Mean age at diagnosis (years)	63.79 (±12.78)	63.63 (±13.44)	63.78 (±12.02)	64.22 (±13.02)	0.969
Tumor stage					
Nonmuscle invasive	184 (80.0)	84 (45.7)	69 (37.5)	31 (16.8)	
Muscle invasive	52 (20.0)	20 (38.5)	22 (42.3)	10 (19.2)	0.653
Tumor grade					
Grade 1	120 (50.8)	52 (43.3)	51 (42.5)	17 (14.2)	
Grade 2	78 (33.1)	39 (50.0)	24 (30.8)	15 (19.2)	
Grade 3	38 (16.1)	13 (34.2)	16 (42.1)	9 (23.7)	0.283
Number					
Single	168 (71.2)	77 (45.8)	64 (38.1)	27 (16.1)	
Multiple	68 (28.8)	27 (39.7)	27 (39.7)	14 (20.6)	0.602
Size					
<3 cm	118 (75.9)	56 (47.5)	42 (35.6)	20 (16.9)	
≥3 cm	118 (24.1)	48 (40.7)	49 (44.4)	21 (14.8)	0.555

**Table 8:** Genotype and allele frequencies of the *TSP-1*-1223 A/G polymorphism among the bladder cancer cases with survival information.

Genotypes	Recurrence (*n* = 78)		Nonrecurrence (*n* = 158)		*P* ^b^	Adjusted HR (95% CI)^b^
	*n*	%	n	%		
AA	25	32.1	79	50.0		1.00 (reference)
AG	33	42.3	58	36.7	0.062	1.68 (0.97–2.88)
GG	20	25.6	21	13.3	**0.002**	**2.63 (1.43–4.83)**

AA	25	32.1	79	50.0		1.00 (reference)
AG + GG	53	67.9	79	50.0	**0.007**	**1.95 (1.20–3.19)**

AG + AA	58	74.4	137	86.7		1.00 (reference)
GG	20	25.6	21	13.3	**0.006**	**2.07 (1.23–3.49)**

A allele	83	53.2	216	63.4		
G allele	73	46.8	100	36.6		*P* ^a^ = 0.001

CI: confidence interval; HR: hazard ratio.

^a^Two-sided *χ* ^2^ test for allele frequencies between the cases and controls.

^b^Adjusted for age, gender, smoking, drinking status, and family history of cancer in cox regression model.

The bold font refers to the statistical significance of result.

## References

[1] Siegel R, Naishadham D, Jemal A (2012). Cancer statistics, 2012. *CA Cancer Journal for Clinicians*.

[2] Liu E, Xiang Y, Jin F (2004). Cancer incidence trends in urban Shanghai, China (1972-1999). *Tumor*.

[3] Kaufman DS, Shipley WU, Feldman AS (2009). Bladder cancer. *The Lancet*.

[4] Rubben H, Lutzeyer W, Fischer N (1988). Natural history and treatment of low and high risk superficial bladder tumors. *Journal of Urology*.

[5] Giovino GA, Mirza SA, Samet JM (2012). Tobacco use in 3 billion individuals from 16 countries: an analysis of nationally representative cross-sectional household surveys. *The Lancet*.

[6] Walz DA (1992). Thrombospondin as a mediator of cancer cell adhesion in metastasis. *Cancer and Metastasis Reviews*.

[7] Taraboletti G, Roberts D, Liotta LA, Giavazzi R (1990). Platelet thrombospondin modulates endothelial cell adhesion, motility, and growth: a potential angiogenesis regulatory factor. *The Journal of Cell Biology*.

[8] Taraboletti G, Roberts DD, Liotta LA (1987). Thrombodspondin-induced tumor cell migration: haptotaxis and chemotaxis are mediated by different molecular domains. *The Journal of Cell Biology*.

[9] Nickoloff BJ, Mitra RS, Riser BL, Dixit VM, Varani J (1988). Modulation of keratinocyte motility. Correlation with production of extracellular matrix molecules in response to growth promoting and antiproliferative factors. *American Journal of Pathology*.

[10] Reed MJ, Puolakkainen P, Lane TF, Dickerson D, Bornstein P, Sage EH (1993). Differential expression of SPARC and thrombospondin 1 in wound repair: immunolocalization and in situ hybridization. *Journal of Histochemistry and Cytochemistry*.

[11] Varani J, Dixit VM, Fligiel SEG (1986). Thrombospondin-induced attachment and spreading of human squamous carcinoma cells. *Experimental Cell Research*.

[12] Roberts DD, Sherwood JA, Ginsburg V (1987). Platelet thrombospondin mediates attachment and spreading of human melanoma cells. *The Journal of Cell Biology*.

[13] Nucera C, Porrello A, Antonello ZA (2010). B-RafV600Eand thrombospondin-1 promote thyroid cancer progression. *Proceedings of the National Academy of Sciences of the United States of America*.

[14] Wei W, Beihua K, Qifeng Y, Xun Q (2010). Hepatocyte growth factor enhances ovarian cancer cell invasion through downregulation of thrombospondin-1. *Cancer Biology and Therapy*.

[15] McElroy MK, Kaushal S, Tran Cao HS (2009). Upregulation of thrombospondin-1 and angiogenesis in an aggressive human pancreatic cancer cell line selected for high metastasis. *Molecular Cancer Therapeutics*.

[16] Zubac DP, Bostad L, Kihl B, Seidal T, Wentzel-Larsen T, Haukaas SA (2009). The expression of thrombospondin-1 and p53 in clear cell renal cell carcinoma: its relationship to angiogenesis, cell proliferation and cancer specific survival. *Journal of Urology*.

[17] Grossfeld GD, Ginsberg DA, Stein JP (1997). Thrombospondin-1 expression in bladder cancer: association with p53 alterations, tumor angiogenesis, and tumor progression. *Journal of the National Cancer Institute*.

[18] Goddard JC, Sutton CD, Jones JL, O’Byrne KJ, Kockelbergh RC (2002). Reduced Thrombospondin-1 at presentation predicts disease progression in superficial bladder cancer. *European Urology*.

[19] Wu X, Lin X, Dinney CP, Gu J, Grossman HB (2007). Genetic polymorphism in bladder cancer. *Frontiers in Bioscience*.

[20] Hannah B-LA, Misenheimer TM, Pranghofer MM, Mosher DF (2004). A polymorphism in thrombospondin-1 associated with familial premature coronary artery disease alters Ca2^+^ binding. *The Journal of Biological Chemistry*.

[21] Zwicker JI, Peyvandi F, Palla R (2006). The thrombospondin-1 N700S polymorphism is associated with early myocardial infarction without altering von Willebrand factor multimer size. *Blood*.

[22] Laherty CD, Gierman TM, Dixit VM (1989). Characterization of the promoter region of the human thrombospondin gene. DNA sequences within the first intron increase transcription. *The Journal of Biological Chemistry*.

[23] Guo N-H, Templeton NS, Al-Barazi H (2000). Thrombospondin-1 promotes *α*3*β*1 integrin-mediated adhesion and neurite- like outgrowth and inhibits proliferation of small cell lung carcinoma cells. *Cancer Research*.

[24] Albo D, Arnoletti JP, Castiglioni A (1994). Thrombospondin (TSP) and transforming growth factor beta 1 (TGF-*β*) promote human A549 lung carcinoma cell plasminogen activator inhibitor type 1 (PAI-1) production and stimulate tumor cell attachment in vitro. *Biochemical and Biophysical Research Communications*.

[25] Yamashita Y, Sendo S, Hosokawa T (1998). Exogenous thrombospondin stimulates the proliferation of non-thrombospondin-producing cells. *International Journal of Oncology*.

[26] Ren B, Yee KO, Lawler J, Khosravi-Far R (2006). Regulation of tumor angiogenesis by thrombospondin-1. *Biochimica et Biophysica Acta*.

[27] Rofstad EK, Graff BA (2001). Thrombospondin-1-mediated metastasis suppression by the primary tumor in human melanoma xenografts. *Journal of Investigative Dermatology*.

[28] Jin RJ, Kwak C, Lee SG (2000). The application of an anti-angiogenic gene (thrombospondin-1) in the treatment of human prostate cancer xenografts. *Cancer Gene Therapy*.

[29] Streit M, Velasco P, Brown LF (1999). Overexpression of thrombospondin-1 decreases angiogenesis and inhibits the growth of human cutaneous squamous cell carcinomas. *American Journal of Pathology*.

[30] Tenan M, Fulci G, Albertoni M (2000). Thrombospondin-1 is downregulated by anoxia and suppresses tumorigenicity of human glioblastoma cells. *Journal of Experimental Medicine*.

[31] Tuszynski GP, Gasic TB, Rothman VL (1987). Thrombospondin, a potentiator of tumor cell metastasis. *Cancer Research*.

[32] Wong SY, Purdie AT, Han P (1992). Thrombospondin and other possible related matrix proteins in malignant and benign breast disease: an immunohistochemical study. *American Journal of Pathology*.

[33] Campbell SC, Volpert OV, Ivanovich M, Bouck NP (1998). Molecular mediators of angiogenesis in bladder cancer. *Cancer Research*.

[34] Agrawal U, Mishra AK, Salgia P, Verma S, Mohanty NK, Saxena S (2011). Role of tumor suppressor and angiogenesis markers in prediction of recurrence of non muscle invasive bladder cancer. *Pathology and Oncology Research*.

[35] Meyers S, Downing JR, Hiebert SW (1993). Identification of AML-1 and the (8;21) translocation protein (AML-1/ETO) as sequence-specific DNA-binding proteins: the runt homology domain is required for DNA binding and protein-protein interactions. *Molecular and Cellular Biology*.

[36] Kim JH, Lee S, Rho JK, Choe SY (1999). AML1, the target of chromosomal rearrangements in human leukemia, regulates the expression of human complement receptor type 1 (CR1) gene. *International Journal of Biochemistry and Cell Biology*.

[37] Tanaka T, Tanaka K, Ogawa S (1995). An acute myeloid leukemia gene, AML 1, regulates hemopoietic myeloid cell differentiation and transcriptional activation antagonistically by two alternative spliced forms. *EMBO Journal*.

[38] Rodríguez-Manzaneque JC, Lane TF, Ortega MA, Hynes RO, Lawler J, Iruela-Arispe ML (2001). Thrombospondin-1 suppresses spontaneous tumor growth and inhibits activation of matrix metalloproteinase-9 and mobilization of vascular endothelial growth factor. *Proceedings of the National Academy of Sciences of the United States of America*.

[39] Gupta K, Gupta P, Wild R, Ramakrishnan S, Hebbel RP (1999). Binding and displacement of vascular endothelial growth factor (VEGF) by thrombospondin: effect on human microvascular endothelial cell proliferation and angiogenesis. *Angiogenesis*.

[40] Ferrara N, Davis-Smyth T (1997). The biology of vascular endothelial growth factor. *Endocrine Reviews*.

[41] Crew JP, O’Brien T, Bradburn M (1997). Vascular endothelial growth factor is a predictor of relapse and stage progression in superficial bladder cancer. *Cancer Research*.

